# Feasibility of Short-Term Redifferentiation in Patients with Radioactive Iodine–Refractory Metastatic Thyroid Cancer

**DOI:** 10.2967/jnumed.125.270055

**Published:** 2025-08

**Authors:** Johannes von Hinten, Oliver Viering, Ralph A. Bundschuh, Feyza Cagliyan, Hermann Wengenmair, Christian H. Pfob, James Nagarajah, Constantin Lapa, Malte Kircher

**Affiliations:** 1 Nuclear Medicine, Faculty of Medicine, University of Augsburg, Augsburg, Germany;; 2 Department of Nuclear Medicine, University of Dresden, Dresden, Germany;; 3 Department of Nuclear Medicine, Pendik Training and Research Hospital, Marmara University, Istanbul, Turkey;; 4 Department of Radiology and Nuclear Medicine, Radboud University Medical Center, Nijmegen, The Netherlands; and; 5 Roentgeninstitut Düsseldorf, Düsseldorf, Germany

**Keywords:** radioiodine-refractory thyroid cancer, redifferentiation, metastatic differentiated thyroid carcinoma, kinase inhibition

## Abstract

Radioactive iodine–refractory thyroid cancer (TC) has a poor prognosis, and restoring iodine uptake is a major therapeutic goal. Recent studies have used tyrosine kinase inhibitors (TKIs) for 3–6 wk to achieve redifferentiation, but preclinical data suggest that maximal effects occur within 8–12 d. **Methods:** In this retrospective study, 8 patients with metastatic radioactive iodine–refractory TC received trametinib plus dabrafenib (for *BRAF*-mutated disease) or trametinib alone (for *BRAF* wild-type disease) for 10 d. Iodine uptake was assessed by ^123^I-scintigraphy; responders received high-dose radioactive iodine therapy, and nonresponders continued TKIs for another 10 d. **Results:** Two patients (both with *BRAF* wild-type disease) achieved successful iodine uptake restoration with significant thyroglobulin reduction after radioactive iodine therapy. Extending TKI treatment to 21 d did not yield further benefit. **Conclusion:** Our pilot study supports preclinical findings that maximal restoration of iodine uptake is achieved after only 10 d of TKI therapy, reducing toxicity and treatment costs. Longer treatment did not provide any additional benefit. Larger prospective trials are needed to confirm these findings.

## Visual Abstract

**Figure:**
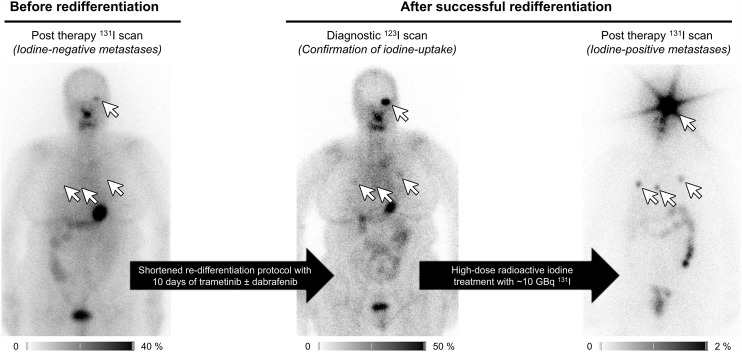

The incidence of thyroid cancer (TC) has increased significantly over the past 3 decades, posing significant health challenges (1). Most differentiated TCs are of follicular origin, with papillary and follicular TC being the most common subtypes. Metastatic disease is the leading cause of TC-related death (2). While radioactive iodine treatment (RAIT) is a key therapy (3–6), 60%–70% of patients with metastatic TC develop radioactive iodine–refractory (RAIR) disease, reducing 10-y survival to approximately 10% (2,7).

Recent studies have shown promising results in restoring iodine uptake in tumor cells by inhibiting the mitogen-activated protein kinase signaling pathway with tyrosine kinase inhibitors (TKIs) (8–11). Weber et al. (9) reported a 35% success rate in a cohort of 20 subjects after 21 d of trametinib and dabrafenib in patients with *BRAF*-mutated disease and trametinib alone in patients with *BRAF* wild-type disease. In 6 of 7 patients who received RAIT, disease stabilization was achieved (9). Leboulleux et al. (12) found that 42 d of treatment with trametinib and dabrafenib led to partial response (38%), stable disease (52%), and progressive disease (10%) in 21 evaluable patients with *BRAF*-mutated, metastatic RAIR TC at 6-mo follow-up.

However, exposure to kinase inhibitors can cause significant side effects, potentially leading to treatment discontinuation. In addition, TKIs are expensive.

Preclinical studies have shown that maximum sodium–iodide symporter restoration to the tumor cell membrane is achieved within 8–12 d of TKI therapy (13). Prolonged use of TKIs did not further improve sodium–iodide symporter expression or iodine uptake; rather, these effects can be diminished as a result of the activation of escape mechanisms (13). This suggests that a shorter duration of TKI treatment could reduce side effects and costs while maintaining therapeutic efficacy.

This study evaluated the efficacy and safety of short-term TKI treatment with trametinib and dabrafenib for restoring iodine uptake in patients with metastatic RAIR TC.

## MATERIALS AND METHODS

All procedures adhered to the Declaration of Helsinki and its later amendments, as well as legal considerations of clinical guidelines. All patients gave written informed consent for imaging and therapy. This study was approved by the Ethics Committee of Ludwig-Maximilians-Universität München (approval ID 24-0213).

Eight consecutive patients (4 women, 4 men; median age, 72 y [range, 65–87 y]) with progressive metastatic RAIR TC underwent redifferentiation treatment in the Department of Nuclear Medicine at the University Hospital of Augsburg from January 2021 to April 2024. RAIR TC was defined according to the 2019 European Thyroid Association guideline (i.e., insufficient radioiodine accumulation in lesions on a therapeutic radioiodine scan or an insufficient decrease in highly sensitive thyroglobulin [hTg] levels after RAIT). All patients had undergone total thyroidectomy and adjuvant RAIT, with some receiving additional treatments, such as further RAIT, surgery, external-beam radiation therapy, or TKI therapy, all of which were completed at least 6 mo before enrollment (Table 1).

**TABLE 1.** Patient Characteristics

Patient No.	Sex	Age (y)	Pathology	Driver mutation	Previous RAIT	Cumulative ^131^I activity (GBq)	Additional therapy after initial TT	Sites of disease	hTg*
1	F	71	PDTC	None	1×	7.4	None	Lung, bone, liver	240
2	M	65	PTC	*BRAF^V600E^*	2×	13.7	Surgery (recurrent local tumor, cervical lymph nodes)	Recurrent local tumor, lung, lymph node	160
3	F	87	Oncocytic	None	2×	11.0	Surgery (liver)	Recurrent local tumor, bone, lymph node, liver	980
4	F	71	PTC	None	1×	5.7	TKI (lenvatinib)	Recurrent local tumor, lung, bone, lymph node, pleura	26,000
5	M	73	Oncocytic	None	1×	3.8	EBRT (mediastinal tumor mass)	Lung, lymph node	840
6	M	68	Oncocytic	None	2×	9.3	None	Recurrent local tumor, lung, bone, lymph node, pleura	9,100
7	F	78	FTC	*NRAS p.Q61K*	5×	46.1	Surgery (skull, lung); brachytherapy (orbita)	Lung, bone, left orbita	590
8	M	84	FTC	None	5×	29.9	Surgery (recurrent local tumor, cervical lymph nodes)	Recurrent local tumor, lung, bone, lymph node	12,000

* Unstimulated hTg before redifferentiation.

TT = total thyroidectomy; PDTC = poorly differentiated thyroid carcinoma; PTC = papillary thyroid carcinoma; EBRT = external-beam radiation therapy; FTC = follicular thyroid carcinoma.

Driver mutations were identified via next-generation sequencing (AmpliSeq Illumina Focus Panel or TruSight Oncology 500 assay; Illumina), examining primary (*n* =1), recurrent (*n* =2), and metastatic (*n* = 5) tumor tissue.

The most recent report from high-dose ^131^I imaging served as the baseline for the presence of iodine-positive or -negative lesions. Periinterventional PET/CT with [^18^F]FDG for baseline staging and assessment of hTg levels was performed under thyroid-stimulating hormone (TSH) suppression.

The redifferentiation protocol was based on the preclinical findings of Nagarajah et al. (13) and performed as described by Weber et al. (9).

Patients received daily TKI treatment for 10 d, either 2 mg of trametinib daily in patients with *BRAF* wild-type disease or a combination of 2 mg of trametinib daily and 75 mg of dabrafenib twice daily in patients with *BRAF*-mutated disease. On day 11, ^123^I restaging under exogenous TSH stimulation determined redifferentiation success, defined as a 2-fold higher iodine uptake in iodine-negative lesions than the mean uptake in healthy liver tissue and a regional tumor-to-background ratio of more than 4, as suggested by Weber et al. (9). In lesions demonstrating successful redifferentiation, RAIT with TSH stimulation was initiated on day 12, with continuation of TKI therapy for an additional 2 d. Nonresponders received another 10-d TKI regimen and returned for reevaluation with scintigraphy on day 21. If successful, RAIT was given on day 22; otherwise, TKI was discontinued.

Safety was monitored at baseline and treatment completion via medical history interview, physical examination, echocardiography, and blood and urine samples. Adverse drug reactions were assessed using the Common Terminology Criteria for Adverse Events, version 5.0.

Dosimetry of iodine uptake by lesions was performed according to the method described by Hänscheid et al. (14), with 1 late-uptake assessment.

Treatment response to RAIT was assessed using hTg levels under TSH suppression and at the first restaging using RECIST 1.1 and PERCIST 1.0. Further details on the materials and methods are provided in the supplemental materials, available at http://jnm.snmjournals.org.

## RESULTS

### Patient Cohort

The most common TC in this patient cohort was oncocytic (*n* = 3), followed by metastatic papillary (*n* = 2), follicular (*n* = 2 ), and poorly differentiated (*n* = 1) TC.

All patients suffered from progressive disease, including local recurrence (*n* = 5) and metastases in the lungs (*n* = 7), lymph nodes (*n* = 6), bone (*n* = 6), liver (*n* = 2), and pleura (*n* = 2).

Mutation analysis was available for all patients, revealing a *BRAF^V600E^* mutation in 1 patient (patient 2) and an *NRAS p.Q61K* mutation in another (patient 7). The remaining patients did not exhibit any driver mutations (Table 1).

The median hTg level at baseline was 840 ng/mL (range, 160–26,000 ng/mL).

### Redifferentiation Treatment

Before redifferentiation, all patients had at least 5 iodine-negative metastases (median, 20; range, 5 to >100). In 4 patients, limited iodine uptake was observed in some lesions, which was insufficient for RAIT; the remaining metastases were iodine-negative. In the remaining 4 patients, none of the tumor manifestations showed iodine accumulation.

**FIGURE 1.**
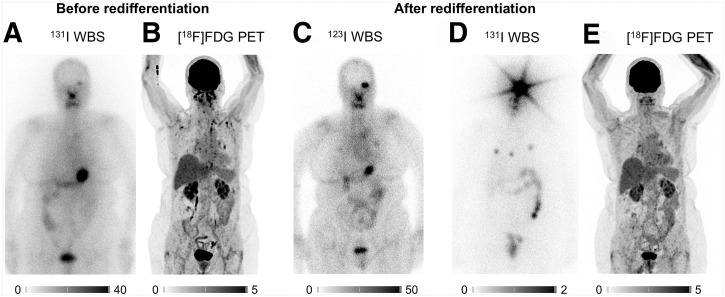
Comparison of baseline ^131^I-scintigraphy, [^18^F]FDG PET (A and B), and postinterventional ^123^I scan (C) after successful redifferentiation in patient 7. Iodine uptake increased, especially in left orbital metastasis and in left and 2 right pulmonary metastases, which led to RAIT. Depiction of whole-body scintigraphy (WBS) scan after high-dose RAIT with significant iodine uptake in newly iodine-avid lesions (D) and of [^18^F]FDG PET scan 4 mo after RAIT (E) with assessment of partial response. Intensity scales are SUV for PET and % for scintigraphic images.

Two patients (patients 7 and 8) demonstrated successful redifferentiation after 10 d of daily TKI treatment, which led to subsequent RAIT (Fig. 1). Two other patients exhibited a slight increase in iodine uptake (most metastases in patient 2 and only the local recurrence in patient 6), but this was not sufficient to justify RAIT. Despite a further 10 d of TKI treatment in these patients, no further increase in iodine uptake was observed in any lesion on day 21. The remaining 4 patients showed no iodine uptake in any lesion on initial ^123^I scintigraphy. Among this group, 2 patients continued TKI treatment, but the second scan provided no evidence of relevant iodine uptake. The remaining 2 patients discontinued TKI treatment due to drug-related side effects (pleural effusion and peripheral edema) (Table 2).

**Table 2.** Course and Efficacy of Redifferentiation Treatment

Patient	SUV_max_ of hottest lesion in baseline [^18^F]FDG PET	Redifferentiation successful after first 10 d of TKI intake?	Redifferentiation successful after 20 d of TKI intake?	CTCAE	Subsequent RAIT
1	9.5	No	No	None	No
2	21.6	No	No	None	No
3	57.1	No	Discontinuation of redifferentiation treatment due to toxicity	3 (peripheral edema)	No
4	59.1	No	Discontinuation of redifferentiation treatment due to toxicity	3 or 4 (pleural effusion, peripheral edema)	No
5	20.1	No	No	2 (skin rash)	No
6	58.8	No	No	2 (skin rash)	No
7	6.4	Yes	NA	None	Yes
8	6.9	Yes	NA	1 (xerostomia)	Yes

CTCAE = Common Terminology Criteria for Adverse Events; NA = not applicable.

### Safety

Three patients did not experience any adverse drug reactions during the 10- or 21-d treatment period. The remaining patients experienced a total of 6 adverse events, 3 of which were classified as grade 1 or 2 (2 cases of grade 2 skin rash and 1 case of grade 1 xerostomia). Two patients discontinued TKI treatment after 10 d due to grade 3–4 pleural effusion (1 patient) and peripheral edema (2 patients) (Table 2). All TKI-related side effects resolved within 3 wk of treatment discontinuation.

### RAIT Efficacy

After successful redifferentiation, RAIT was administered orally (9.9 GBq of ^131^I in patient 7 and 11.7 GBq of ^131^I in patient 8). The mean tumor-absorbed dose per administered activity was 2.70 ± 0.35 Gy/GBq, with maximum absorbed doses of 18.0 Gy in patient 7 and 17.1 Gy in patient 8 (Supplemental Table 1).

After redifferentiation and RAIT, the hTg level in patient 7 decreased from 590 ng/mL to 53 ng/mL after 4 mo. In patient 8, the hTg level dropped from 12,000 ng/mL to 6,800 ng/mL within 5 wk and subsequently increased and exceeded the original baseline level after 17 wk. In contrast, patient 7 experienced only a slight rise in hTg level after treatment, with levels reaching 62 ng/mL at the last documented follow-up 13 mo after RAIT, well below the baseline value.

**FIGURE 2.**
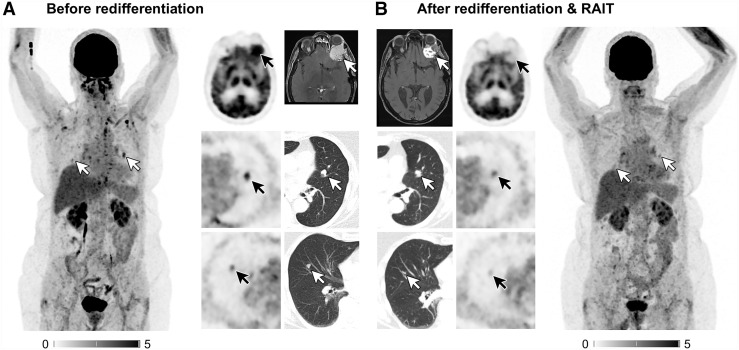
Comparison of glycolytic activity and MRI or CT morphologic course of osseous and pulmonary lesions of patient 7 before (A) and after (B) redifferentiation with consecutive RAIT with significant decrease of [^18^F]FDG uptake and reduction of lesion volumes (only pulmonary lesions and orbita lesion) with assessment of partial response 4 mo after RAIT. hTg level decreased from 590 ng/mL to 53 ng/mL during same time frame in patient 7. Intensity scales are SUV.

Patient 7 achieved partial (metabolic) response according to PERCIST 1.0 and RECIST 1.1 (Fig. 2). Patient 8 exhibited stable disease.

## DISCUSSION

Long-term TKI therapy, while a mainstay for treatment of RAIR TC, carries a significant burden of side effects, highlighting the need for alternative strategies (12).

By restoring iodine uptake through short-term TKI therapy for RAIR TC, repeat RAIT, which may increase the low 10-y survival rate of 10%, reduce morbidity by decreasing the metastatic burden, and delay the need for kinase inhibition, is feasible (8,11).

Although previous studies mainly used redifferentiation approaches that included 3–6 wk of TKI therapy, the ideal treatment protocol is under debate. In fact, preclinical studies have demonstrated that the highest degree of iodine incorporation may be achieved after 8–12 d of TKI use (13), which may translate to lower costs and fewer adverse events in the clinical setting (9,12).

In contrast to Weber et al. (9), we defined RAIR TC on the basis of the 2019 European Thyroid Association guidelines, which, in addition to iodine-negative imaging reports, also consider the biochemical response to RAIT. This allowed us to include patients with inadequate hTg decline after RAIT with iodine-positive metastases.

The literature suggests that mutated *NRAS* may be a favorable factor for successful iodine restoration. This mutation was found in 1 of 2 responding patients.

Successful redifferentiation and RAIT led to a significant decrease in serum tumor marker levels of 91% and 43% and correlated with partial (metabolic) response and stable disease in first-response assessment imaging in patients 7 and 8, respectively.

Importantly, extending TKI treatment to 21 d did not improve redifferentiation rates in our cohort. This suggests that the benefits of TKI-induced redifferentiation may plateau within the first 10 d and that prolonged exposure does not necessarily lead to increased iodine uptake. While 2 patients’ lesions remained completely iodine-negative at the time of the second ^123^I scan, the other 2 patients’ lesions demonstrated only a slight increase in iodine uptake, which was practically unchanged compared with the findings from their first ^123^I scan. These findings support the preclinical results reported by Nagarajah et al. (13) and may indicate that prolonged TKI treatment does not enhance the success rate of redifferentiation.

In our cohort, 6 adverse events were observed in 5 patients (63%), half of which were grade 1 or 2 skin rash and xerostomia. However, grade 3–4 pleural effusion and peripheral edema prompted discontinuation of TKI treatment after 10 d in 2 patients. Although this rate is still lower than the overall 96% side-effect rate reported by Leboulleux et al. (12), who noted 7 grade 3 or 4 adverse events in 24 patients, the potential for severe adverse events may favor the short-term use of TKI in redifferentiation protocols. However, given the preliminary nature of this small retrospective analysis, further research to determine the optimal redifferentiation protocol is clearly needed.

Our study had significant limitations. Foremost, the small patient population and retrospective design preclude firm conclusions. For the 2 patients with successful redifferentiation undergoing RAIT, no pretherapeutic dosimetry to tailor administered activity of ^131^I was performed to optimize achievable tumor-absorbed doses, leading to the use of standard high activities.

Furthermore, the short follow-up period (up to 4 mo) hampers the evaluation of disease stabilization and thus the therapeutic benefit of redifferentiation and subsequent RAIT, and we cannot provide any information on potential long-term toxicity. Finally, mutation status was assessed only in a single lesion per patient, potentially missing *BRAF* mutations in lesions with discordant molecular profiles. However, a high concordance regarding *BRAF* mutation status between primary tumor and metastases in TC has been described (15,16).

In summary, short-term redifferentiation in metastatic RAIR TC after only 10 d of daily TKI therapy is feasible, reducing side effects and overall treatment costs. Longer TKI treatment did not improve the effects of redifferentiation. Further prospective, randomized controlled trials are warranted to compare the efficacy and safety of this short-term TKI redifferentiation protocol with established, longer-term protocols, stratifying patients based on mutation status. A design of a possible prospective trial is provided in Supplemental Figure 1. Future research should also incorporate pretherapeutic dosimetry to tailor the administered activity of ^131^I after redifferentiation, aiming to optimize tumor-absorbed doses and improve treatment outcomes.

## CONCLUSION

Our pilot study demonstrated that short-term (10-d) TKI therapy is a feasible approach for restoring iodine uptake in selected patients with metastatic RAIR TC, potentially reducing side effects and treatment costs. These results support preclinical data suggesting that optimal redifferentiation is achieved within 8–12 d, with no additional benefit from longer treatment. Larger prospective trials to confirm these findings and optimize patient selection and treatment protocols are warranted.

## DISCLOSURE

No potential conflict of interest relevant to this article was reported.

## Notes

KEY POINTS **QUESTION:** Is the restoration of iodine uptake in patients with RAIR TC using short-term (10-d) oral TKIs feasible? **PERTINENT FINDINGS:** Successful restoration of tumor iodine uptake after only 10 d of TKI use was achieved in 2 of 8 patients with RAIR TC. Prolongation of treatment for up to 21 d did not achieve further redifferentiation in any subject. **IMPLICATIONS FOR PATIENT CARE:** Short-term redifferentiation in RAIR TC after only 10 d of TKI therapy is feasible, reducing side effects and overall costs. Further prospective trials in larger patient cohorts are warranted.
